# Global water quality changes posing threat of increasing infectious diseases, a case study on malaria vector *Anopheles stephensi* coping with the water pollutants using age-stage, two-sex life table method

**DOI:** 10.1186/s12936-022-04201-x

**Published:** 2022-06-08

**Authors:** Mahmoud Fazeli-Dinan, Mostafa Azarnoosh, Mehmet Salih Özgökçe, Hsin Chi, Nasibeh Hosseini-Vasoukolaei, Farzad Motevalli Haghi, Mohamad Ali Zazouli, Seyed Hassan Nikookar, Reza Dehbandi, Ahmadali Enayati, Morteza Zaim, Janet Hemingway

**Affiliations:** 1 Department of Medical Entomology and Vector Control, Health Sciences Research Center, Faculty of Health, Mazandaran University of Medical Sciences, Sari, Iran; 2 Faculty of Agriculture, Department of Plant Protection, Van Yuzuncu Yil University, 65080 Van, Turkey; 3 Institute of Applied Ecology, Fujian Agriculture and Forestry University, Fuzhou, 350002 China; 4 Department of Environmental Health, Health Sciences Research Center, Addiction Institute, Faculty of Health, Mazandaran University of Medical Sciences, Sari, Iran; 5 Environment Technologies Research Center, Ahvaz Jundishapour University of Medical Sciences, Ahvaz, Iran; 6 Department of Medical Entomology and Vector Control, School of Public Health and National Institute of Health Research, Tehran University of Medical Sciences, Tehran, Iran; 7 Department of Vector Biology, Liverpool School of Tropical Medicine, Liverpool, UK

**Keywords:** Global water pollutants, *Anopheles stephensi*, Malaria vector, Life table, Adaptation

## Abstract

**Background:**

Water pollution due to uncontrolled release of chemical pollutants is an important global problem. Its effect on medically important insects, especially mosquitoes, is a critical issue in the epidemiology of mosquito-borne diseases.

**Methods:**

In order to understand the effect of water pollutants on the demography of *Anopheles stephensi*, colonies were reared in clean, moderately and highly polluted water for three consecutive generations at 27 °C, 75% RH, and a photoperiod of 12:12 h (L:D). The demographic data of the 4th generation of *An. stephensi* were collected and analysed using the age-stage, two-sex life table.

**Results:**

The intrinsic rate of increase (*r*), finite rate of increase (*λ*), mean fecundity (*F*) and net reproductive rate (*R*_0_) of *An. stephensi* in clean water were 0.2568 d^−1^, 1.2927 d^−1^, 251.72 eggs, and 109.08 offspring, respectively. These values were significantly higher than those obtained in moderately polluted water (*r* = 0.2302 d^−1^, λ = 1.2589 d^−1^, 196.04 eggs, and *R*_0_ = 65.35 offspring) and highly polluted water (*r* = 0.2282 d^−1^, λ = 1.2564 d^−1^, 182.45 eggs, and *R*_0_ = 62.03 offspring). Female adult longevity in moderately polluted (9.38 days) and highly polluted water (9.88 days) were significantly shorter than those reared in clean water (12.43 days), while no significant difference in the male adult longevity was observed among treatments.

**Conclusions:**

The results of this study showed that *An. stephensi* can partially adapt to water pollution and this may be sufficient to extend the range of mosquito-borne diseases.

## Background

Mosquitoes are important vectors of many diseases, including malaria, dengue fever, chikungunya, yellow fever, Zika, encephalitis, and filariasis [1–3]. *Anopheles* mosquitoes are particularly important as they transmit malaria [4]. The World Health Organization (WHO) estimated there were 241 million malaria cases globally and 627,000 deaths in 2020 which were only slightly lower than those in 2010 and 2017 [5–7]. In Iran, *Anopheles stephensi* is an important vector of malaria [8, 9]. This species is widely distributed in Asia including India, Pakistan, Afghanistan, Iran, Iraq, Bahrain, Oman, Saudi Arabia, Bangladesh, Myanmar, and South China [10]. Recently the distribution of this species expanded to Sri Lanka, Djibouti, Ethiopia, Sudan and Somalia [11]. In Iran, this species has a wide distribution in Khuzestan, Fars, Kerman, Hormozgan, Sistan va Baluchestan, and southern Kermanshah provinces [8, 12, 13]. It has been the main malaria vector in Iran for many decades and still causes sporadic malaria cases in the South and Southeast areas of Iran [8].

Malaria transmission by *Anopheles* mosquitoes is influenced by their population density, which is affected by a number of abiotic factors of aquatic habitats [14]. The physicochemical factors of aquatic habitats affect the survival, development, and fecundity of *Anopheles* and, consequently can play a key role in malaria outbreaks [15–17]. Usually, *Anopheles* mosquitoes breed in clean water bodies; however, some *Anopheles* larvae are able to complete their life cycle in polluted waters [18, 19]. Human domestic, industrial and agricultural activities have increased pollution in aquatic environments through wastewater, solid waste, application of detergents, pesticides and fertilizers [20, 21]. Global warming and environmental changes may increase aquatic pollution in the future [22–24]. There is the potential for adaptation of mosquitoes to these pollutants [25]. Therefore, the assessment of the impact of water pollutants on the population fitness of *Anopheles* is important [26, 27].

The ecological features of vector populations such as stage-specific survival, development, and fecundity are key components of disease epidemiology [28]. Life tables are used to evaluate the effect of water pollution on the growth, development, survival and fecundity of mosquitoes [29]. This offers the most comprehensive understanding of the population characteristics [30, 31], and female age-specific life tables have been widely used in numerous studies. These have been applied to studies of the survivorship and reproductive strategies of colonized culicine and anopheline mosquitoes such as *Aedes aegypti* [32], *Culex quinquefasciatus* [33], *Culex tritaeniorhynchus* [34], *An. stephensi* and *Anopheles culicifacies* [30]. Because the female age-specific life tables ignore the male population and cannot describe the stage differentiation, they cannot be used to predict population growth. Huang and Chi [35], Huang et al. [36] and Chi et al. [37] discussed in detail the problems of the female age-specific life tables. In contrast to the female age-specific life table, the age-stage, two-sex life table can describe the stage differentiation and include the male population [29, 38, 39].

The goal of the present study was to compare the demographic characteristics of *An. stephensi* using the age-stage, two-sex life table approach in clean, moderately, and highly polluted water to assess the adaptive capacity of *An. stephensi* in breeding sites containing different degrees of pollutions, and provide detailed information on life table data of *An. stephensi* in polluted water.

## Methods

### *Anopheles stephensi* strain

The beech laboratory strain of *An. stephensi* was colonized in 1947 [40]. This subsection describes the methods used to maintain the colony in the insectary at the Medical Entomology and Vector Control Department, Mazandaran University of Medical Sciences, where it has been maintained for at least five generations. Eggs of *An. stephensi* (no older than 24 h) were put in white enamel trays (38 cm in length, 25 cm in width, and 10 cm in height) containing two litres of tap water allowing no more than 200 larvae in each tray [41]. Trays were kept at 27 ± 2 °C, 75 ± 5% RH and a photoperiod 12:12 h (L: D). About 24 h after eggs hatching, 0.5 g of TetraMin® fish food was daily added to the tray as larval food [41, 42]. When the 4^th^ instars were observed, trays were checked daily for the presence of pupae. Pupae were collected and transferred into screened plastic cups containing tap water. Emerged adults, with a 1:1 ratio of male and female (25 pairs), were transferred to a screened rearing cage (40 × 40 × 40 cm) where they could readily mate. A restrained guinea pig was supplied to females for blood-feeding for 120 min. Initially, attempts were made to sedate the guinea pigs with different drugs, however this caused unusual malformations in the legs of the emerging adult mosquitoes (becoming hook like). Unfortunately, a high proportion failed to fully hatch from the pupal case and drowned. Mosquito blood feeding was carried out according to the ethical approval No. IR.MAZUMS.REC.1398.1398.

A vial containing cotton wicks soaked in a 10% sugar solution was placed in each cage as the source of energy for adults. To minimize the growth of mold or bacteria, sugar solution and cotton wicks were replaced every three days. Mosquitoes were offered blood meals every 3 days, until all females had died. A white enamel bowl (7 cm diameter × 15 cm height) filled with 150 ml tap water was placed in the cage to collect mosquito eggs. The bowl was checked daily and newly laid eggs were transferred to trays filled with tap water.

### Pollutants and preparation of polluted water samples

In this study, total organic carbon (TOC), nitrate, and phosphate which are the common contaminants of aquatic environments were considered as the pollutants. Water samples of three different qualities were prepared: clean water treatment (i.e., control treatment), moderately polluted water, and highly polluted water. Deionized water (DI) and tap water were used for the preparation of all stock solutions and polluted water. Clean water was prepared with tap water and deionized water (1:1 in ratio). All tap waters used for preparation of treatment waters were set aside for 24 h in the laboratory to remove chlorine. The chemical characteristics of moderately and highly polluted water samples containing different concentrations of nitrate, phosphate and total organic carbon (TOC) which were prepared in the laboratory are listed in Table 1. The moderately polluted water mimicked the concentration of pollutants in mosquito larval habitats in Mazandaran Province [16]. Highly polluted water had the maximum permissible amount of each component in drinking water according to the World Health Organization standard [43]. Water samples were prepared by adding humic acid, sodium nitrate (NaNO_3_) and potassium dihydrogen phosphate (KH_2_PO_4_) to clean water as the sources for TOC, nitrate and phosphate, respectively [44].

**Table 1:** Quality of clean, moderately polluted and highly polluted water: concentration of phosphate, nitrate, and total organic carbon (TOC), pH and electrical conductivity (EC)

Parameter	Clean water	Moderately polluted water	Highly polluted water
Phosphate (mg. L^−1^)	< 0.01	2	6
Nitrate (mg. L^−1^)	< 0.01	5	50
TOC (mg. L^−1^)	< 0.01	5	10
pH	7.33	7.17	7.24
EC (µS/cm)	380.4	465.6	544.2

Total organic carbon (TOC) as a measure of the amount of organic compounds in all water samples was measured according to the Standard Method 5310B using TOC-VCSH TOC Analyzer (Shimadzu, Japan). If a water sample contains gross solids or insoluble matter, it is filtered to obtain homogenous solution. After this preparatory step, sample is injected into the analyzer according to the manufacturer’s instructions. Organic carbon is oxidized (during a combustion process in the instrument) to carbon dioxide, CO_2_. The resulting CO_2_ is purged from the solution and carried to an infrared analyzer specifically tuned to quantify the CO_2_ contents of the sample. The instrument’s microprocessor converts the detector signal to organic carbon concentration in mg/L based on a stored calibration curve [45]. The concentration of nitrate and phosphate was determined by spectrophotometry (Hach Company DR 2000), according to the Standard Methods [46]. Electrical conductivity (EC) and pH were measured using a table pH/EC meter (Eutech Instrument). The concentration of candidate chemicals along with pH and EC in the different water samples were controlled every three days throughout the experiments.

### Life table study

Age-stage, two-sex life tables were used to analyse the influence of water conditions on the development and survival of *An. stephensi*. The mosquitoes were raised in the three different water treatments for four generations before recording differences in the population parameters. The three water treatments were: clean, moderately and highly polluted water.

To conduct the study, newly emerged male (n = 25) and female (n = 25) adults were released into screened rearing cages (40 × 40 × 40 cm) and were offered 10% sucrose solution in vials with cotton wicks. Mosquitoes were blood-fed after mating on a restrained guinea pig. White enamel bowls (7 cm diameter × 15 cm height) filled with 150 ml of the respective water were used to collect mosquito eggs. To determine the life table, newly hatched first instar larvae (L_1_) were used. For this purpose, egg masses (about 200 eggs) laid within 24 h of each treatment were transferred to white enamel bowls containing each of the water treatments. After hatching, 150 newly hatched first instar larvae (L_1_) were individually transferred into 8-cell containers (one larva per cell; dimension of each cell: 10 × 3 × 3 cm) containing 35 ml of each water type per cell. Larvae were fed daily with a diet of TetraMin® fish food (0.0025 gr per larva). The numbers of live mosquitoes at each developmental stage [47] were observed daily. Subsequently, newly emerged males and females were reared in group (as many as emerged in circa 1:1 ratio) in cages (40 × 40 × 40 cm) and data files were prepared as group-reared life table and analysed [48]. A white paper sheet was placed on the bottom of the cage to facilitate the identification and removal of dead mosquitoes. Dead adults were sexed and removed daily. Both sugar solution and blood meal were routinely offered to the mosquitoes as detailed previously. A white enamel bowl filled with water of the respective treatment was provided for egg collection. The bowl was replaced daily. Egg masses were kept until hatched and the number of hatched eggs was recorded as daily fecundity. The procedure was repeated until all adults in the cage died. During the life table study, mosquitoes were kept at 27 ± 2 °C, 75 ± 5% RH and a photoperiod 12:12 h (L: D).

### Life table data analyses

All life table raw data on the development, survival and daily fecundity of *An. stephensi* were analysed according to the age-stage, two-sex life table theory [38, 39] using the TWOSEX-MSChart program [49]. The age-stage-specific survival rate (*s*_*xj*_: the probability that a newborne egg will survive to age *x* and stage *j*); the age-specific survival rate (*l*_*x*_: probability that a newborne egg will survive to age *x*); female fecundity (*F*: eggs/female); the age-stage-specific fecundity (*f*_*xj*_: the number of hatched eggs produced by female adult at age *x*); the age-specific fecundity (*m*_*x*_: the number of eggs per individual at age *x*); and age-specific maternity (*l*_*x*_*m*_*x*_: the product of *l*_*x*_ and *m*_*x*_) were calculated. All population parameters including the intrinsic rate of increase (*r*), the finite rate of increase (λ), the net reproductive rate (*R*_0_) and the mean generation time (*T*) were calculated.

The age-stage-specific survival rate (*s*_*xj*_) was calculated as:

$${S}_{xj}=\frac{{n}_{xj}}{{n}_{01}}$$

The age-specific survival rate (*l*_*x*_) was calculated for both females and males using the following formula:

$$l_{x} = \sum\limits_{{j = 1}}^{\delta } {s_{{xj}} }$$

where *δ* is the last stage of the cohort.

The age-specific fecundity (*m*_*x*_) at age *x* was calculated as:

$${m}_{x}=\frac{\sum_{j=1}^{\delta }{s}_{xj}{f}_{xj}}{\sum_{j=1}^{\delta }{s}_{xj}}$$

The net reproductive rate (*R*_0_) is the sum of all *l*_*x*_*m*_*x*_ (age-specific maternity) which considers the survival rate and is calculated by the following equation:

$$R_{0} = \sum\limits_{{x = 0}}^{\infty } {l_{x} m_{x} }$$

The intrinsic rate of increase (*r*) is calculated using the Lotka-Euler equation with age indexed from zero [50] using the following formula:

$$\sum\limits_{{x = 0}}^{\infty } {e^{{ - r(x + 1)}} l_{x} m_{x} = 1}$$

The finite rate of increase (λ) was calculated as λ = *e*^*r*^. The mean generation time (*T*) is the time length that a population requires to increase to *R*_0_-fold of its size as time approaches infinity and the population settles down to a stable age-stage distribution and is calculated as:

$$T=\frac{{\mathrm{ln}R}_{0}}{r}$$

Age-stage specific life expectancy (*e*_*xj*_) defined as the number of days that an individual of age *x* and stage *j* is expected to live, is calculated using the following equation by assuming ${s}_{xj}^{^{\prime}}=1$;

$$e_{{xj}} = \sum\limits_{{i = x}}^{\infty } {\sum\limits_{{y = j}}^{\delta } {s_{{iy}}^{\prime } } }$$

where ${s}_{iy}^{^{\prime}}$ is the probability that an individual of age *x* and stage *j* will survive to age *i* and stage *y*.

The reproductive value (*v*_*xj*_) represents the contribution of individuals of age *x* and stage *j* to the future population [51] and was estimated using the following formula [29, 52]:

$$v_{{xj}} = \frac{{e^{{r(x + 1)}} }}{{s_{{xj}} }}\sum\limits_{{i = x}}^{\infty } {e^{{ - r(x + 1)}} } \sum _{{y = j}}^{\delta } s^{\prime } _{{{\text{iy}}}} {\text{f}}_{{{\text{iy}}}}$$

Since anopheline mosquitoes require 8–12 days (depending on the malaria parasite species) to become infective [53, 54], the number of female adults that survived more than 10 days (*N*_*f*,AL≥10_) and the proportion of these female adults in the life table cohort (*N*_*f*,AL≥10_/*N*) were calculated.

To estimate standard errors of the life-table parameters, group-reared data was converted to individual-reared data [48]. Then the bootstrap technique was used with 100,000 resampling [55, 56] and differences between treatments were assessed using the paired bootstrap [57, 58].

The parameter of adult pre-oviposition period (APOP) was calculated based on the period between the emergence of an adult female until initiation of the first oviposition; and total pre-oviposition period (TPOP) was calculated based on the time interval from egg to the beginning of oviposition of the resulting adult. Oviposition days (*O*_*d*_) defined as the number of days in which females laid eggs [58, 59].

### Population projection

The life table data obtained were used to project the population size of *An. stephensi* [36]. Ten newly laid eggs of *An. stephensi* were used as the initial population to project the total population size for 40 days using the computer program TIMING-MSChart (Chi 2021). The 100,000 bootstrap results of the finite rate (λ) were sorted to find the life table of 2.5th and 97.5th percentiles (i.e., the 2500th and 97,500th sorted bootstrap samples) of the finite rate. These life tables were then used to predict the variability of population growth [36].

## Results

### Development, survival and reproduction

The durations of all stages of *An. stephensi* reared in three water treatments are shown in Table 2. The effect of water quality on developmental duration of the egg, larva, pupa, and adult stage of *An. stephensi* were variable. The longevity of adult females raised in clean water (average of 12.43 ± 0.81 days) was significantly longer than those whose immatures were raised in moderately (9.38 ± 0.65 days) or highly (9.88 ± 0.60 days) polluted water. There were significant differences in pupal duration among treatments. The shortest pupal duration was observed in the clean water treatment. However, there was no significant difference in total pre-adult duration among treatments. The immature survival rate in clean water (0.79% ± 0.03) was significantly higher than that in highly polluted water (0.64% ± 0.04) (Table 2).

**Table 2:** Developmental duration, longevity, adult pre-oviposition period (APOP), total pre-oviposition period (TPOP), oviposition days (*O*_*d*_), total longevity of all eggs, and mean fecundity (± SE) of *Anopheles stephensi* reared in different water treatments

Stage	Clean water		Moderately polluted		Highly polluted	
	*n*	Mean ± SE	*n*	Mean ± SE	*n*	Mean ± SE
Egg (days)	150	1.11 ± 0.03 b	150	1.14 ± 0.03 ab	150	1.19 ± 0.03 a
1^st^ Instar (days)	149	1.88 ± 0.03 a	150	1.63 ± 0.04 b	150	1.80 ± 0.03 a
2^nd^ Instar (days)	147	2.20 ± 0.06 a	150	2.12 ± 0.03 a	149	2.09 ± 0.04 a
3^rd^ Instar (days)	127	1.94 ± 0.06 a	143	1.94 ± 0.05 a	120	2.00 ± 0.06 a
4^th^ Instar (days)	121	2.28 ± 0.01 b	121	1.90 ± 0.04 ab	108	2.24 ± 0.04 a
Pupa (days)	118	1.21 ± 0.04 c	104	2.02 ± 0.01 a	96	1.35 ± 0.05 b
Pre-adult (days)	118	10.52 ± 0.05 a	104	10.42 ± 0.05 a	96	10.50 ± 0.05 a
Immature survival rate (%)	150	0.79 ± 0.03 a	150	0.69 ± 0.04 ab	150	0.64 ± 0.04 b
Male adult longevity (days)	53	3.45 ± 0.14 a	54	3.56 ± 0.19 a	45	3.89 ± 0.20 a
Male total longevity (days)	53	13.98 ± 0.18 a	54	13.83 ± 0.23 a	45	14.33 ± 0.26 a
Female adult longevity (days)	65	12.43 ± 0.81 a	50	9.38 ± 0.65 b	51	9.88 ± 0.60 b
Female total longevity (days)	65	22.94 ± 0.86 a	50	19.96 ± 0.71 b	51	20.43 ± 0.66 b
Total longevity (days)	150	16.64 ± 0.61 a	150	14.57 ± 0.43 b	150	14.33 ± 0.48 b
APOP (days)	63	1.48 ± 0.06 a	48	1.40 ± 0.07 a	50	1.44 ± 0.07 a
TPOP (days)	63	12.00 ± 0.00 a	48	12.00 ± 0.00 a	50	12.00 ± 0.00 a
Oviposition days (*O*_*d*_) (days)	65	7.84 ± 0.57 a	48	5.90 ± 0.46 b	50	6.06 ± 0.44 b
Female fecundity (*F*) (eggs/female)	65	251.72 ± 23.38 a	50	196.04 ± 24.03 ab	51	182.45 ± 18.95 b

Means in the same row followed by different lower-case letters are significantly different based on the confidence intervals of the differences between different treatments using the paired bootstrap test (*P* < 0.05)

Means in the same column followed by different upper-case letters denote significant difference between sexes in the same treatment

When *An. stephensi* was reared in clean water, the mean fecundity was 251.72 ± 23.38 eggs/female, which is the highest among all treatments and significantly higher than that of highly polluted water (182.45 ± 18.95 eggs/female, *P* = 0.021), but not significantly different from that in the moderately polluted water (196.04 ± 24.03 eggs/female, *P* = 0.095). In the clean water treatment, the oviposition days (*O*_*d*_: the number of oviposition days) of *An. stephensi* was 7.84 ± 0.57 days, which was significantly longer than those in moderately and highly polluted treatments (Table 2).

**Fig. 1:**
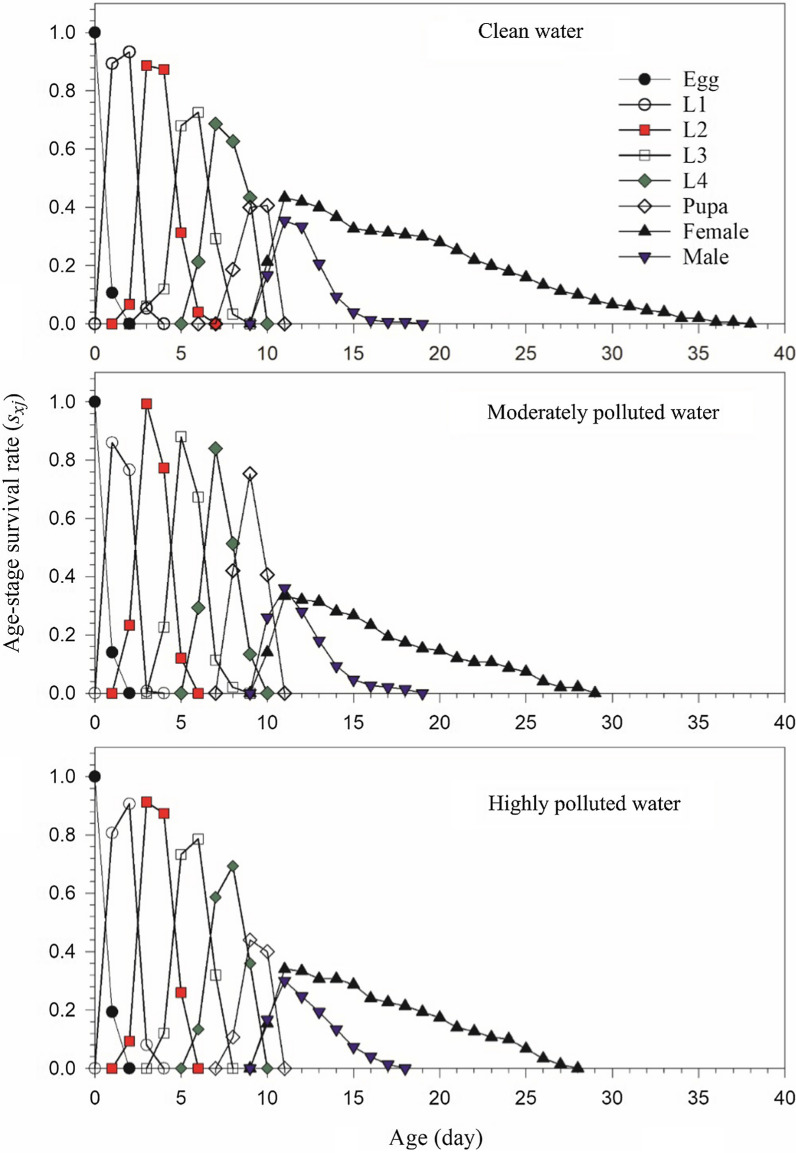
Age-stage survival rate curves (*s*_*xj*_) of *Anopheles stephensi* in clean, moderately and highly polluted water treatments

There were no significant differences in adult pre-oviposition period (APOP) and total pre-oviposition period (TPOP) among treatments. The average adults’ female longevity was 12.43 ± 0.81 days in the clean water treatment, which was significantly longer than those in moderately (9.38 ± 0.65 days) and highly polluted water (9.88 ± 0.60 days). The total longevity of female individuals in clean water treatment was also significantly longer than those in moderately and highly polluted treatments. The age-stage-specific survival rates (*s*_*xj*_) (Fig. 1) showed that untreated *An. stephensi* survived longer than those in the moderately and highly polluted water. The shorter *s*_*xj*_ curves of male *An. stephensi* in comparison with those of females showed longer survival of females. There were no significant differences in male adult longevity and total male longevity among treatments.

**Fig. 2:**
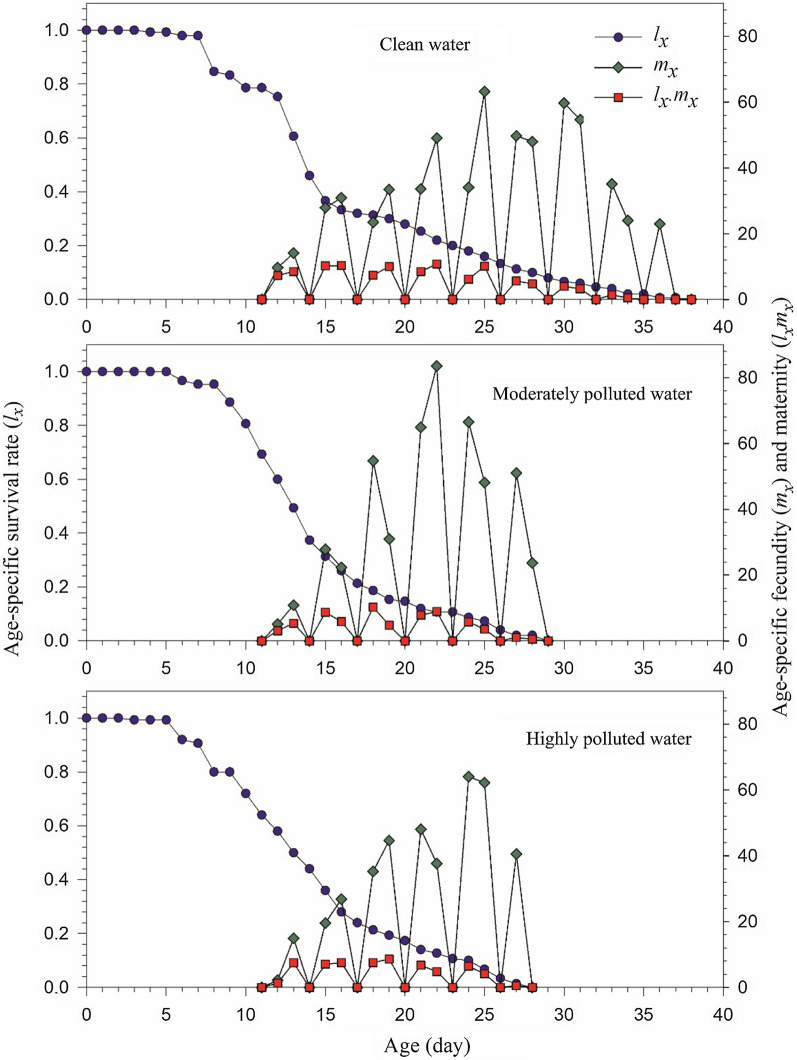
Age-specific survival rate (*l*_*x*_), age-specific fecundity (*m*_*x*_), and age-specific maternity (_*lx*_*m*_*x*_) of *Anopheles stephensi* in clean, moderately and highly polluted water treatments

Age-specific survival (*l*_*x*_), age-specific fecundity of the total population (*m*_*x*_), and age-specific maternity (*l*_*x*_*m*_*x*_) of *An. stephensi* are demonstrated in Fig. 2. The *m*_*x*_ curve revealed the periodic reproduction of *An. stephensi*. The *m*_*x*_ curve for *An. stephensi* reared in clean water was extended from age 12 d to 38 d, which was significantly longer than the other two treatments. The longer reproductive period in the clean water treatment was consistent with the higher number of oviposition days (*O*_*d*_ = 7.84 ± 0.57) (Table 2). Although higher peaks in the *m*_*x*_ curve was observed in the moderately polluted water, the net maternity (*l*_*x*_*m*_*x*_) was lower than that of the clean water treatment due to the lower survival rate (*l*_*x*_).

### Population parameters

The intrinsic rate of increase (*r*), finite rate of increase (*λ*), the net reproductive rate (*R*_0_) and mean generation time (*T*) are given in Table 3. The value of *r*, λ and *R*_0_ for *An. stephensi* in clean water was 0.2568 ± 0.0066 d^−1^, 1.2927 ± 0.0085 d^−1^ and 109.08 ± 14.33 offspring/individual, respectively. These values were higher than those in moderately and highly polluted water. No significant differences were observed between moderately and highly polluted water. There was no significant difference in the mean generation time (*T*) among treatments.

**Table 3:** Means (± SE) of population parameters of *Anopheles stephensi* reared in clean, moderately and highly polluted water: *r*, intrinsic rate of increase; λ, finite rate of increase; *R*_0_, net reproductive rate; *T*, mean generation time; *N*_*f*,AL≥10_, number of female adults with adult longevity ≥ 10 days; *N*_*f*,AL≥10_/*N*, proportion of female adults in cohort with adult longevity ≥ 10 days

Parameters	Clean water	Moderately polluted	Highly polluted
*r* (d^−1^)	0.2568 ± 0.0066 a	0.2302 ± 0.0081 b	0.2282 ± 0.0079 b
*λ* (d^−1^)	1.2927 ± 0.0085 a	1.2589 ± 0.0102 b	1.2564 ± 0.0098 b
*R*_0_ (offspring/individual)	109.08 ± 14.33 a	65.35 ± 10.91 b	62.03 ± 9.47 b
*T* (d)	18.27 ± 0.24 a	18.16 ± 0.28 a	18.08 ± 0.23 a
*N*_*f*,AL≥10_	45.00 ± 5.62 a	22.00 ± 4.14 b	27.00 ± 4.68 b
*N*_*f*,AL≥10_/*N*	0.30 ± 0.0375 a	0.1467 ± 0.0288 b	0.18 ± 0.0312 b

Means in the same row followed by different letters are significantly different based on the confidence intervals of the differences between treatments using the paired bootstrap test (*P* < 0.05)

Out of 150 eggs, 45 female adults survived longer than 10 days (*N*_*f*,AL≥10_ = 45.00 ± 5.62 females) in clean water, which was significantly more than that in moderately polluted water (*N*_*f*,AL≥10_ = 22.00 ± 4.14 females) and that in highly polluted water (*N*_*f*,AL≥10_ = 27.00 ± 4.68 females). The proportion of these female adults in clean water was 0.3 (i.e., *N*_*f*,AL≥10_/*N* = 0.3 ± 0.0375), which was significantly greater than that in the moderately polluted water (*N*_*f*,AL≥10_/*N* = 0.1467 ± 0.0288), and highly polluted water (*N*_*f*,AL≥10_/*N* = 0.18 ± 0.0312), respectively.

**Fig. 3:**
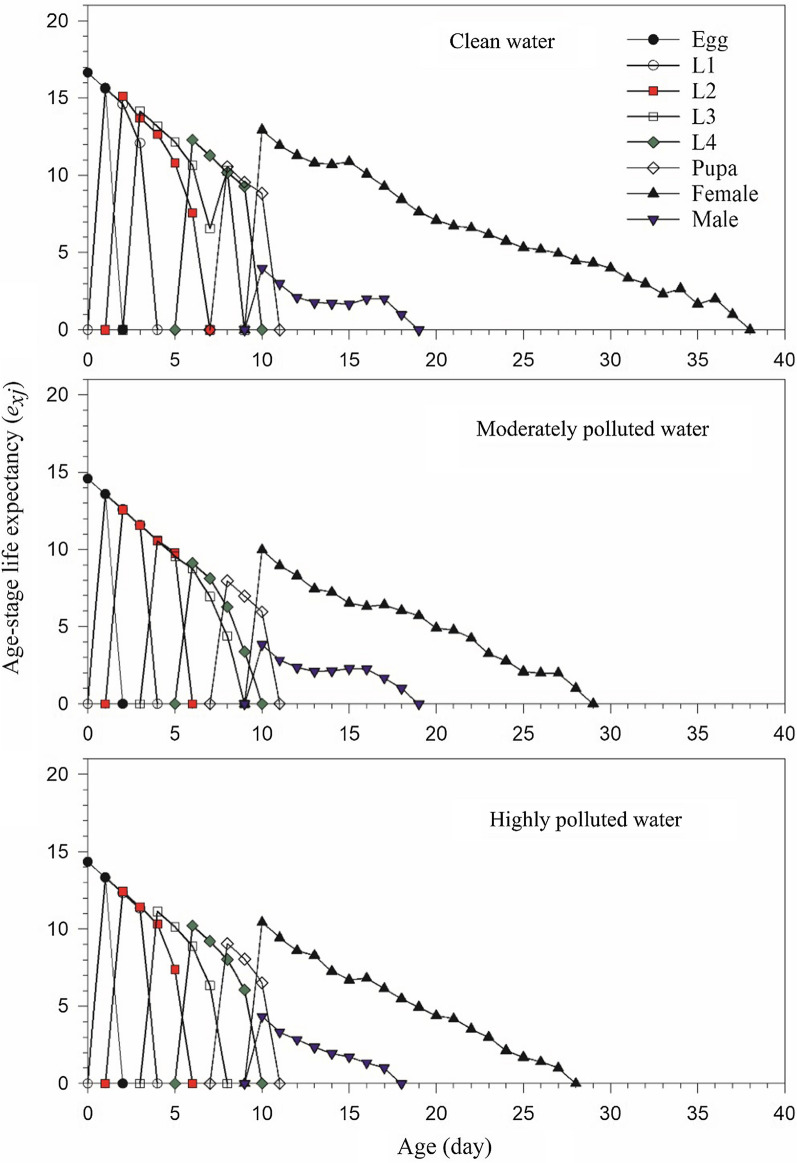
Age-stage-specific life expectancy (*e*_*xj*_) of *Anopheles stephensi* in clean, moderately and highly polluted water treatments

**Fig. 4:**
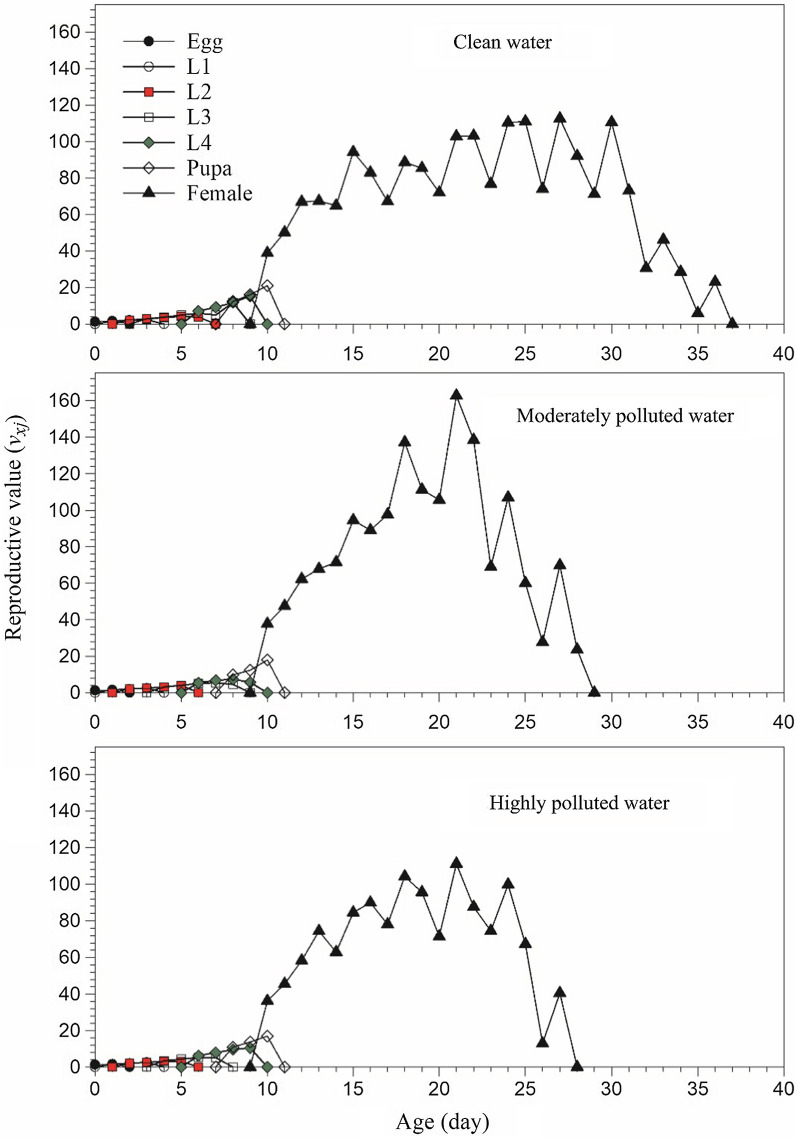
Age-stage-specific reproductive value (*v*_*xj*_) of *Anopheles stephensi* in clean, moderately and highly polluted water treatments

The life expectancy (*e*_*xj*_) of each age-stage group of *An. stephensi* is illustrated in Fig. 3. It represents the time that an individual of age *x* and stage *j* is expected to live [60]. The mean life expectancy of a newly laid egg (*e*_01_) was 16.64, 14.57 and 14.33 d in clean, moderately and highly polluted water treatment, respectively (Table 2). When the first female adult emerged, life expectancy was 12.94, 9.96 and 10.43 d in the clean, moderately and highly polluted water (Fig. 3), respectively; while the life expectancy curve for the male adult began with 3.93, 3.83 and 4.33 d in the clean, moderately and highly polluted water, respectively. The lower curves of male adults showed the male adults had a shorter longevity than females. The reproductive value (*v*_*xj*_) is the contribution of individuals of age *x* and stage *j* to the future population (Fig. 4). At age 0, the reproductive value *v*_01_ is exactly the finite rate of increase (λ), i.e., 1.2927 ± 0.0085, 1.2589 ± 0.0102 and 1.2564 ± 0.0098 d^−1^, in the clean, moderately and highly polluted water, respectively. The first peak of reproductive value for *An. stephensi* reared in clean, moderately and highly polluted water was 38.88, 37.75, and 36.22 d^−1^ at age 10 d when the female adult emerged.

### Population projection

**Fig. 5:**
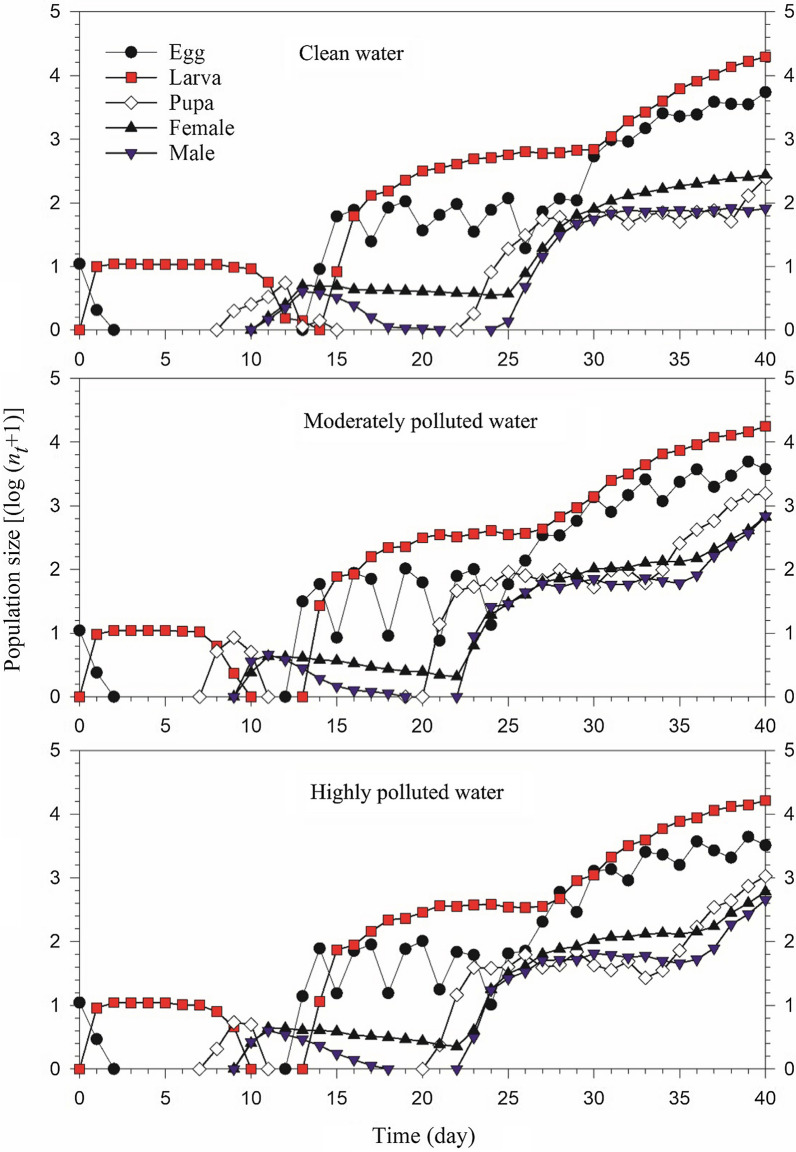
Population projection of *Anopheles stephensi* in clean, moderately and highly polluted water treatments

**Fig. 6:**
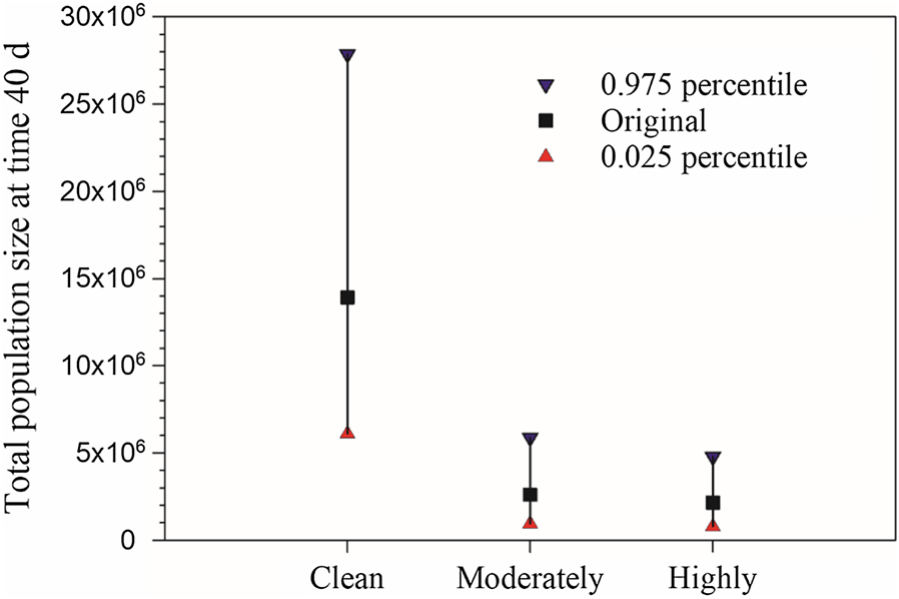
Population growth projection and the 0.025 and 0.975 confidence intervals of *Anopheles stephensi* in clean, moderately and highly polluted water treatments

Population growth projections with stage structures are shown in Fig. 5. The stage overlaps during population growth are evident. The total population size at the end of 40 days and its uncertainty are shown in Fig. 6. In the clean water treatment, the population size reached 63,246 individuals after 40 days, it was much higher than those in the moderately polluted water (24,401 individuals) and highly polluted water treatments (21,732 individuals), respectively (Fig. 6).

**Fig. 7:**
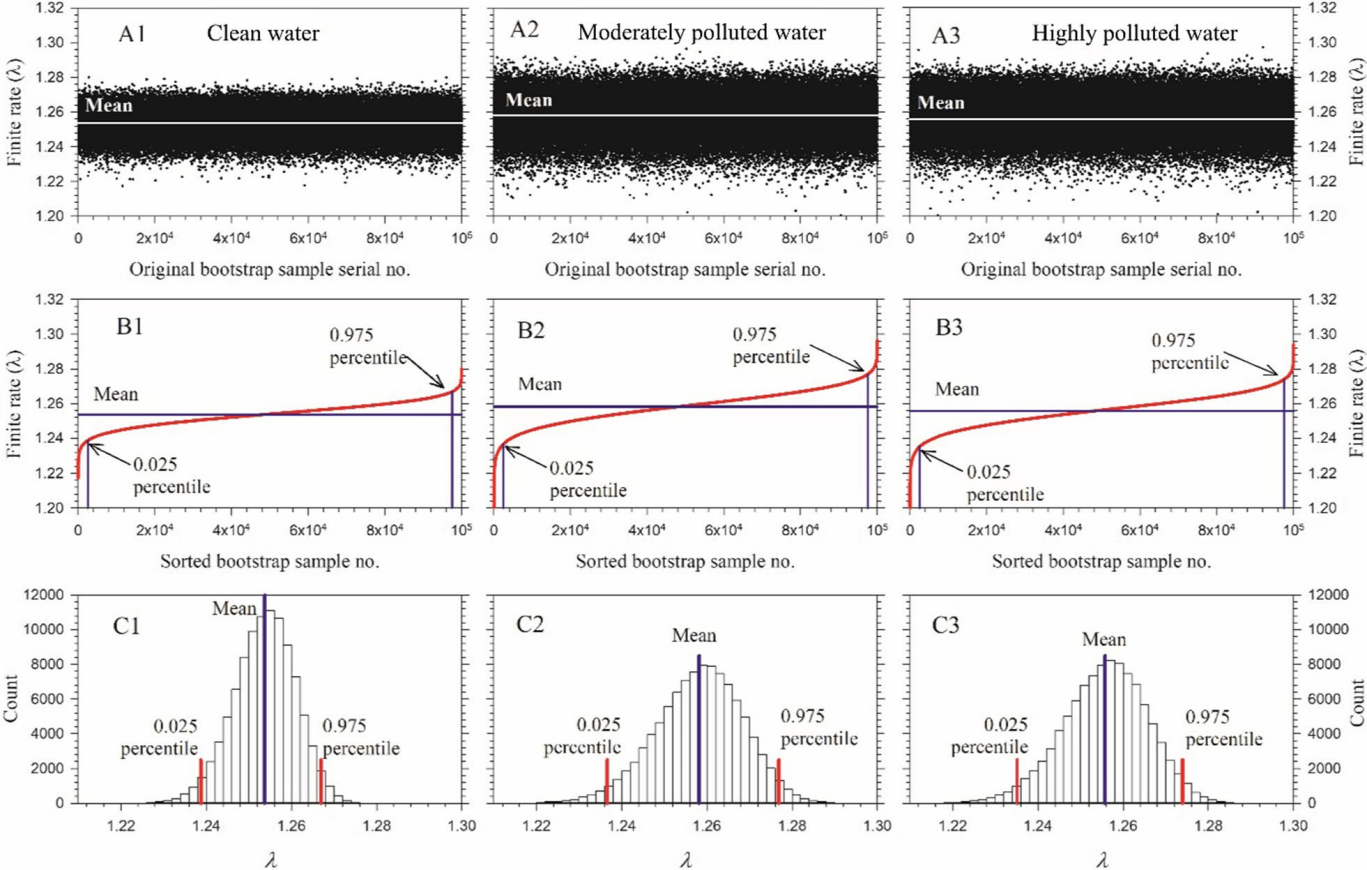
Top figures **A1**–**A3** The unsorted finite rates of increase of 100,000 bootstrap results of the *Anopheles stephensi* in clean, moderately and highly polluted water treatments. The finite rates of 100,000 fluctuated randomly around the mean. Middle figures **B1**–**B3** The 100,000 finite rates of increase were sorted in ascending order. Bottom figures **C1**–**C3** The histogram of 100,000 finite rates. Finite rates of 0.025 and 0.975 percentiles can be observed in (**B1**–**B3**) and (**C1**–**C3**)

By using the bootstrap technique, the uncertainty of population parameter of *An. stephensi* was estimated by using 100,000 resampling. The uncertainty of finite rate was shown in Fig. 7. The 100,000 finite rates randomly fluctuated around the mean (Fig. 7A1–A3). When these values were sorted in ascending order, a smooth curve was obtained (Fig. 7B1–B3). Frequency distribution of the 100,000 bootstrap finite rates of the *An. stephensi* in the different water habitats showed a normal distribution (Fig. 7C1–C3). The 0.025 and 0.975 percentiles of the finite rate of increase were obtained from the sorted data (Fig. 7B1–B3, C1–C3).

## Discussion

In this study, the effect of water pollution on the population fitness of *An. stephensi* is presented*.* The mosquito larvae of malaria vectors can adapt to aquatic environments with different physicochemical characteristics [25, 61–63]. *Anopheles* species, such as *An. stephensi*, *An. culicifacies*, *Anopheles gambiae* and  *Anopheles coluzzii* can adapt to a wide range of water bodies including waste or polluted waters [14, 25, 64, 65]. Knowledge on the population fitness of major malaria vectors breeding in different aquatic environments can inform disease and vector management strategies. Life table data offers comprehensive information about a population, and provide a useful tool for population ecology and pest management [36]. Based on the age-stage, two-sex life table, it was demonstrated that *An. stephensi* could successfully survive and reproduce when their eggs and larvae were reared in moderately or highly polluted water. Using Kaplan–Meier estimator with log-rank test, Oliver and Brooke showed similar adaptation of *Anopheles arabiensis* in metal polluted water [27]. Because the Kaplan–Meier method is only a descriptive method for the survival rate while ignoring the fecundity, it offers limited information about the population fitness.

In the present study, there were no significant differences in the development time of the total pre-adult duration of *An. stephensi* among clean, moderately polluted, and highly polluted water. However, the mean longevity of female adults emerging from clean water (12.43 d) was significantly longer than those from the moderately (9.38 d) and highly polluted water (9.88 d). It is necessary to mention here that female adult longevity influences vector potential, because the malaria parasite requires time to complete their development and sporogonic life cycle inside the mosquito body [28, 53]. This usually takes 8–12 days (intrinsic incubation period – the time required for an individual female to become infectious) depending on the malaria parasite species and the environmental factors [53, 54]. This means that at least some of the individuals in the cohorts may live longer than the intrinsic incubation period required for the sporogonic life cycle.

Despite significant differences in female longevity, there was no difference in adult pre-oviposition (APOP) and total pre-oviposition period (TPOP), i.e. the time needed to begin oviposition did not change due to water pollution. Earlier studies showed that *An. gambiae* and *Aedes aegypti* could adapt to water pollutants including cadmium chloride, copper nitrate and lead nitrate with costs of biological fitness such as reduction of egg viability, immature survivorship and reduced reproductive capacity [27, 66].

The life table parameters (*r*, *λ*, and *R*_0_) of *An. stephensi* were reduced in polluted water compared with those in clean water. It was revealed that *An. stephensi* has the ability to adapt to increasing levels of nitrate, phosphate and TOC, despite the unfavourable effect on the mosquito’s life table parameters.

However, the adaptation of mosquitoes to polluted water poses a serious challenge to the spread of diseases, such as malaria [66, 67], but the biological costs to mosquitoes should not be overlooked. For a correct assessment of the cost of biological fitness for adaptation, it is necessary to consider multiple population characteristics, as with the age-stage, two-sex life table, which includes the development duration, stage differentiation, survival and daily fecundity [38, 39]. After four generations of selection, the costs for mosquitoes to adapt to polluted water was largely seen as reduced longevity of the adult females, which consequently resulted in a lower number of oviposition events and lower lifetime fecundity. Limited studies have been performed on the underlying molecular response of *Anopheles* to water pollutants, e.g., tolerance to heavy metals can be caused by differential expression of metallothioneins and mucins [68, 69]. It was suggested that mosquitoes can regulate their biological program through proteome changes to counter polluted aquatic habitats [68]. Some species of *Anopheles*, such as *An*. *gambiae,* could genetically adapt to chemically altered aquatic habitats [66]. Also, previous studies showed that the life history parameters of mosquitoes are affected by ecological features, therefore they may undergo changes (molecular/structural) in order to adapt to the new conditions [28, 70]. Reduced fecundity can be the cost to tolerate pollutants in aquatic habitats [28, 66], as in this study. This adaptation is important for *An. stephensi,* which prefers to live near its human hosts and, therefore, increasingly encounters chemical pollutants in potential breeding sites, especially in urban areas.

The decreased life table parameters of *Anopheles* in polluted water did cause reduction in the rate of population growth. However, the long-term effect of mosquitoes' adaptation to water pollution needs to be monitored. First of all, pollution can affect the biodiversity of the aquatic organisms so that the species composition changes from natural species to compatible species [71]. Therefore, if natural enemies of the mosquito do not tolerate pollutants, they will lose the capacity of natural reduction of the mosquito population. Secondly, adaptation of *Anopheles* mosquitoes to polluted water can predispose them to insecticide resistance selection [72–74]. Therefore, chemical control programmes may fail to reduce the mosquito population. Some pollutants, such as nitrates and phosphates, can create a large proliferation of algae and bacteria, which is generally detrimental to aquatic organisms. This does not seem to be a serious problem for the *Anopheles* because their larvae and pupae breath directly from air through their siphons [75, 76], so an increase in the growth of algae and bacteria may increase mosquito larval food sources [77–79]. Hence although there is a measurable difference in life table parameters of mosquitoes in polluted water under laboratory conditions, these may be mitigated in field conditions.

The importance of this issue is understood by considering the fact that malaria incidence rate and its management can be assessed by the mathematical model such as "basic reproductive rate" (*R*_*0*_) [80] which has been used for the malaria risk assessment. *R*_*0*_ is calculated using the ${R}_{0}=\frac{m{a}^{2}bc{p}^{n}}{r(-lnp)}$ formula [80]. This is very important to note that changes in the life table parameters affect the density (*m*) and probability of living (*p*) of *Anopheles* mosquitoes, the two most important components of the disease reproduction rate (*R*_*0*_). According to the results of this study, the reduced life table parameters will result in the reduction of the parameters of m and p in the above equation; therefore, it seems that the disease transmission and risk of malaria would reduce. However, care must be taken to extend the negative impact of life table parameters under laboratory conditions on the *R*_*0*_ to the real life situation, and natural field condition studies with the same or other pollutants are recommended to conclude the impact of the water pollutants on the risk of malaria spread in a given area.

It should be noted that the present study faced with some limitations including: for individual rearing of mosquitoes, eggs would have to be singly put in small rearing trays, during this procedure, damage to the eggs might have been inevitable, the end result of which have been observed by rather small hatching rate. Therefore, the study was performed on individual L_1_ stage. Secondly, individual rearing of male and female adults was not possible in cages because most females did not show a desire to feed blood or if they did, they laid a small amount of eggs; therefore, this part of the study was performed as a group.

## Conclusion

Findings presented in this paper demonstrate the impact of environmental pollutants including nitrate, phosphate and TOC on life table parameters such as *r* and *R*_*0*_, among others, of *An. stephensi* in the laboratory. Larval stages of *An. stephensi* prefer unpolluted water [25, 53–55], however, in the face of increasing environmental pollution on one hand and climate change leading to alteration of weather patterns (droughts, floods) on the other, they evolve to adapt to the situation enduring some fitness costs. Therefore, this adaptation may impact the distribution range of *An. stephensi*, as well as changes in the vectorial capacity of the mosquito, factors that may have epidemiological and vector control implications.

## Data Availability

The datasets analysed during the current study are available from the corresponding author (aenayati1372@gmail.com) on request.

## Abbreviations

*r*: The intrinsic rate of increase
*λ*: Finite rate of increase
*R*_0_: The net reproductive rate
*T*: Mean generation time
*e*_*xj*_: Life expectancy
*s*_*xj*_: Age-stage-specific survival rate
*f*_*xj*_: Age-stage-specific fecundity
*l*_*x*_: Age-specific survival rate
*m*_*x*_: Age-specific fecundity
*l*_*x*_*m*_*x*_: Age-specific maternity
APOP: Adult pre-oviposition period
TPOP: Total pre-oviposition period
